# A cytogenetic analysis with chromosome painting in typical antbirds (Passeriformes, Thamnophilidae) and an inference on chromosome evolution in Passeriformes

**DOI:** 10.1007/s10577-026-09812-7

**Published:** 2026-09-21

**Authors:** Talita Fernanda Augusto Ribas, Cleusa Yoshiko Nagamachi, Melquezedec Luis Pinheiro, Patricia Caroline Mary O´Brien, Malcolm Andrew Ferguson- Smith, Fengtang Yang, Alexandre Aleixo, Julio Cesar Pieczarka

**Affiliations:** 1 Laboratório de Citogenética, GEBEM, Centro de Estudos Avançados da Biodiversidade, Instituto de Ciências Biológicas, Universidade Federal do Pará, Parque de Ciência e Tecnologia do Guamá, Avenida Perimetral da Ciência, Km 01, Terreno 11, CEP: 66075-750 Belém, Pará Brasil; 2 Laboratório de Genética e Biodiversidade, Instituto de Ciências da Educação, Universidade Federal Do Oeste Do Pará, UFOPA, Santarém, Pará Brasil; 3 Cambridge Resource Centre for Comparative Genomics, Department of Veterinary Medicine, University of Cambridge, Cambridge, UK; 4 5Cytogenetics Facility, Wellcome Trust Sanger Institute, Hinxton, UK; 5 School of Life Sciences and Medicine, Shandong University, Jinan, China; 6 Instituto Tecnológico Vale/ITV, Belém, PA 66055-200 Brazil

**Keywords:** Amazon, Birds, Fission–fusion, Ancestral Karyotype, ZOO-FISH

## Abstract

The typical antbirds (Passeriformes, Thamnophilidae) are a monophyletic group comprising 225 species in 45 genera, primarily distributed in the lowland forests of the Neotropics. There is limited karyotypic information for this bird family, including chromosome morphology and diploid number (2n), highlighting the importance of understanding the evolutionary history of karyotypic diversification of this family. Few species in Thamnophilidae have been karyotyped to date, with observed diploid numbers ranging from 76 to 82 chromosomes. This study examined the karyotype of three Thamnophilidae species using chromosome painting: *Phlegopsis nigromaculata*, *Thamnophilus aethiops*, a*nd Pyriglena leuconota*. Whole chromosome probes from *Burhinus oedicnemus* (BOE) and *Gallus gallus* (GGA) were used, and results were compared with existing data. Additionally, three more species (*Myrmotherula longipennis*, *Myrmoborus myotherinus*, and *Thamnomanes caesius*) were analyzed using G-banding and/or Giemsa staining. All karyotypes aligned with the standard Aves and Passeriformes karyotype, displaying high diploid numbers (2n = 80 in *Pyriglena leuconota,* 2n = 82 in *Thamnophilus aethiops*, and 2n = 86 in *Phlegopsis nigromaculata*), few macrochromosomes, and numerous microchromosome pairs. Furthermore, our analysis with GGA probes in Thamnophilidae species corroborates the data observed in other Passeriformes families, indicating that the centric fission of GGA2 is a characteristic of Suboscines. Based on this result, we propose an ancestral karyotype for Suboscines that features the fission of GGA-2, which may offer a more robust explanation for the karyotypic diversity found among birds of the order Passeriformes.

## Introduction

Thamnophilidae, commonly referred to as typical antbirds, constitute a diverse group of insectivorous Passeriformes, with approximately 200 species documented in Brazil (de Piacentini 2015). These birds are categorized as either obligate ant-followers, which exclusively employ this foraging strategy, or professional ant-followers, which utilize it opportunistically (Willis & Oniki 1978). The seven genera recognized as obligate ant-followers are *Phaenostictus*, *Pithys*, *Willisornis*, *Gymnopithys*, *Rhegmatorhina*, *Phlegopsis*, and *Oneillornis* (sensu Isler et al. 2014). This evolutionary specialization represents a form of parasitism and is considered relatively recent within Neotropical history (Brumfield et al. 2007). Given that obligate ant-followers rely on limited resources and face heightened competition for food, they are at a greater risk of extinction compared to their professional counterparts (Koh et al. 2004), which underscores the urgent need to understand their genetic and chromosomal diversity.

Currently, cytogenetic data for the Thamnophilidae family are limited, with karyotypes available for only five species: *Pyriglena leucoptera* (2n = 82; Ledesma et al. 2002), *Isleria hauxwelli* (2n = 80; Lucca 1974), *Thamnophilus doliatus* (2n = 82; Lucca and Chamma 1977), *Dysithamnus mentalis* (2n = 76; Lucca 1974), and *Willisornis vidua* (2n = 80; Ribas et al. 2021*).* Moreover, literature indicates that some Thamnophilidae birds are classified as polytypic taxa (Fernandes et al. 2013; Isler and Whitney 2011; Maldonado-Coelho et al. 2013; Aleixo et al. 2009), which may imply a higher number of species than currently catalogued (Mata et al. 2009; Miyaki 2013; Carneiro et al. 2012; Sousa-Neves et al. 2013). These traits make the Thamnophidae birds adequate to investigate chromosomal evolution in birds.

However, classic evolutionary karyotypic studies often employ G-banding, which offers limited information for avian chromosomes compared to mammalian chromosomes. This limitation stems from less distinct macrochromosome bands in birds and low microchromosome band resolution due to their small size (Griffin et al. 2007). Consequently, the use of Fluorescence *in situ* Hybridisation (FISH) in avian species has been effective for karyotypic analysis (Kretschmer et al. 2018b), allowing for a more detailed investigation of chromosomal rearrangements that G-banding cannot resolve.

Although passerines generally have a stable diploid number, with 2n = 80 and karyotypes with similar macrochromosomes, chromosome painting studies show that this group has a complex pattern of pericentric and paracentric inversions, involving chromosomes homologous to GGA1q, both in oscines and suboscines (Kretschmer et al. 2014; dos Santos et al. 2015). Moreover, the fission of GGA-2 remains a subject of ongoing debate: it is unclear whether this rearrangement represents a synapomorphy for Suboscines as a whole (Ribas et al. 2021) or is restricted to the parvorder Furnariida (de Oliveira et al. 2020). To address this question, the present study aims to investigate chromosomal evolution within the Thamnophilidae, thereby testing the validity of these competing hypotheses regarding the origin of GGA-2 fission.

Despite the large number of existing Passeriformes (~ 6000 species), where just a few have been investigated by chromosome painting to date (Nanda et al. 2011; Kretschmer et al. 2014, 2015, 2018a, b; dos Santos et al. 2015; Rodrigues et al. 2018), highlighting the extreme scarcity of cytogenetic studies in this Order. Building upon the scarce data available for this family, a study on *Willisornis vidua* (WVI) utilized *Burhinus oedicnemus* (BOE) and *Gallus gallus* (GGA) set probes, as well as GGA and *Taeniopygia guttata* (TGU) BAC chromosomes (Ribas et al. 2021). This research identified a fusion event between a microchromosome and a macrochromosome (GGA-17 and GGA-5), along with several rearrangements such as inversions and translocations between GGA and WVI. To expand these findings and further investigate chromosomal evolution in Thamnophilidae birds, we used whole chromosome probes from *Burhinus oedicnemus* (BOE) and *Gallus gallus* (GGA) to analyze the karyotypes of three genera: *Phlegopsis*, *Thamnophilus*, and *Pyriglena* and G-banding or Giemsa to *Myrmotherula longipennis, Myrmoborus myotherinus*, and *Thamnomanes caesius* wich were not analysed based on cytogenetics yet, from the Amazon Forest in Brazil. The rationale for using both probe sets is twofold: because GGA is considered close to the Putative Ancestral Karyotype (PAK), and BOE has one of the lowest chromosome numbers among birds (Nie et al. 2009), using them together allows for a broader and more comparative examination of chromosomal evolution in these species, combining an ancestral reference with a highly rearranged one.

## Material and methods

### Samples and chromosomal preparation

Specimens of Thamnophilidae birds were collected from natural populations in the Brazilian Amazon regions of Belém, Tapajós, Rondônia, and Xingu endemism areas (Table 1). Bone marrow preparations were carried out following colchicine treatment (Garnero and Gunski 2000). Voucher specimens were deposited in the bird collection of Museu Paraense Emilio Goeldi, Pará, Brazil. JCP holds a permanent field permit number 13248 from “Instituto Chico Mendes de Conservação da Biodiversidade”. The Cytogenetics Laboratory from UFPa has special permits 19/2003 for sample transport and 52/2003 for research use, granted by the Brazilian Ministry of Environment. The Ethics Committee (Comitê de Ética Animal da Universidade Federal do Pará) approved this research (Permit 68/2015). Specimens were kept in the lab with food and water, maintained under controlled conditions, until euthanasia was performed using an intraperitoneal injection of buffered and diluted barbiturates after local anesthesia.

**Table 1:** Species of Thamnophilidae collected and analysed with cytogenetic methods

Species	Sex	Locality	Coordenates	WCP	G-banding or Giemsa
*Thamninophilus aethiops*	1 F 1 M 1 M and 1F	Santa Bárbara Juruti Flona Tapajós	1° 12′ 14″ S/48° 17′ 39″ W 02º09′08"S/56º05′32"W 2° 24′ 05″ S/55° 04′ 40″ W	Yes	Yes
*Phlegopsis nigromaculata*	1 F 1 F	Flona Tapajós Mocajuba	2° 24′ 05″ S/55° 04′ 40″ W 2°39′53.7''S/49°35''18.3''W	Yes	Yes
*Pyriglena leuconota*	1 M 1 M	Mocajuba Rio Maria	2°39′53.7''S/49°35''18.3''W 07º18′38"S/50º02′54"W	Yes	Yes
*Myrmotherula longipennis*	1 M	Santa Bárbara	1° 12′ 14″ S/48° 17′ 39″ W	No	Yes
*Myrmoborus myotherinus*	1 F	Juruti	02º09′08"S/56º05′32"W	No	Yes
*Thamnomanes caesius*	1 M	Flona Tapajós	2° 24′ 05″ S/55° 04′ 40″ W	No	Yes

*WCP* Whole Chromosome Painting

### Fluorescence *in Situ* Hybridisation (FISH)

Chromosome painting was performed using GGA and BOE whole chromosome probes as described by Nie et al. (2009). The probes were produced by isolating chromosomes via flow cytometry at the Cambridge Resource Centre for Comparative Genomics, Department of Veterinary Medicine, University of Cambridge, UK. Primary DOP-PCR products of whole sorted chromosomes were amplified and labeled. Metaphase chromosomes were captured using a CCD camera (AxioCam MR monochrome) connected to a Zeiss Axiophot and controlled by Axiovision 3.0 software. Chromosomal nomenclature followed Levan et al. (1964), and the chromosomes were measured using the software Image J 1.48 k. Some GGA probes did not work on our sample, so correlations between the WCP of BOE and GGA were made in some situations (Nie et al. 2009).

## Results

The results of chromosome painting are summarized in Table 2.

**Table 2:** Chromosomal correspondence among *Burhinus oedicnemus*, *Gallus gallus*, *Glyphorynchus spirurus* (Ribas et al. 2018), *Thamnophilus aethiops*, *Phlegopsis nigromaculata*, *Pyriglena leuconota* (present work) and *Willisornis vidua* (Ribas et al. 2021), revealed by FISH with BOE chromosome-specific paints

BOE 2 N = 42	GGA 2 N = 78	GSP 2 N = 82	TAE 2 N = 82	PLE 2 N = 80	PNI 2 N = 82	WVI 2 N = 80
1	1	3, 4	1, 1a	1, 1a	1, 1a	1, 1a
2	2	2, 5, W	3, 3a	3, 3a	3, 3a	3, 3a
3	3	1	2	2	2	2
4	4q	6	4	4	4	4
5	7, 8	7, 11	7, 8	7, 8	7, 8	7, 9
6	5	8, W	5	5	5	6
7	9, 2 micros	9	6	6	6	8, 9
8	4p, 1 micro	4p, 1 micro	4a	4a	4a	2 micros
9	6	2 micros	10	10	10, 11	7p, 1 micro
10	2 micros	2 micros	2 micros	2 micros	2 micros	2 micros
11	2 micros	2 micros	2 micros	2 micros	3 micros	5pd, 1 micro
12	2 micros	2 micros	2 micros	2 micros	2 micros	2 micros
13	2 micros	2 micros	2 micros	2 micros	2 micros	6pd, 7pd, 1 micro
14	2 micros	2 micros	2 micros	2 micros	2 micros	2 micros
15 + 16	3 micros	3 micros	3 micros	3 micros	3 micros	3 micros
17 + 18 + 19 + 20	1 micro	3 micros	4 micros	4 micros	4 micros	4 micros
Z	Z	Z, W, 1 micro	ZZ	ZW	ZW	Z, W, 1 micro

### Karyotype description

**Fig. 1:**
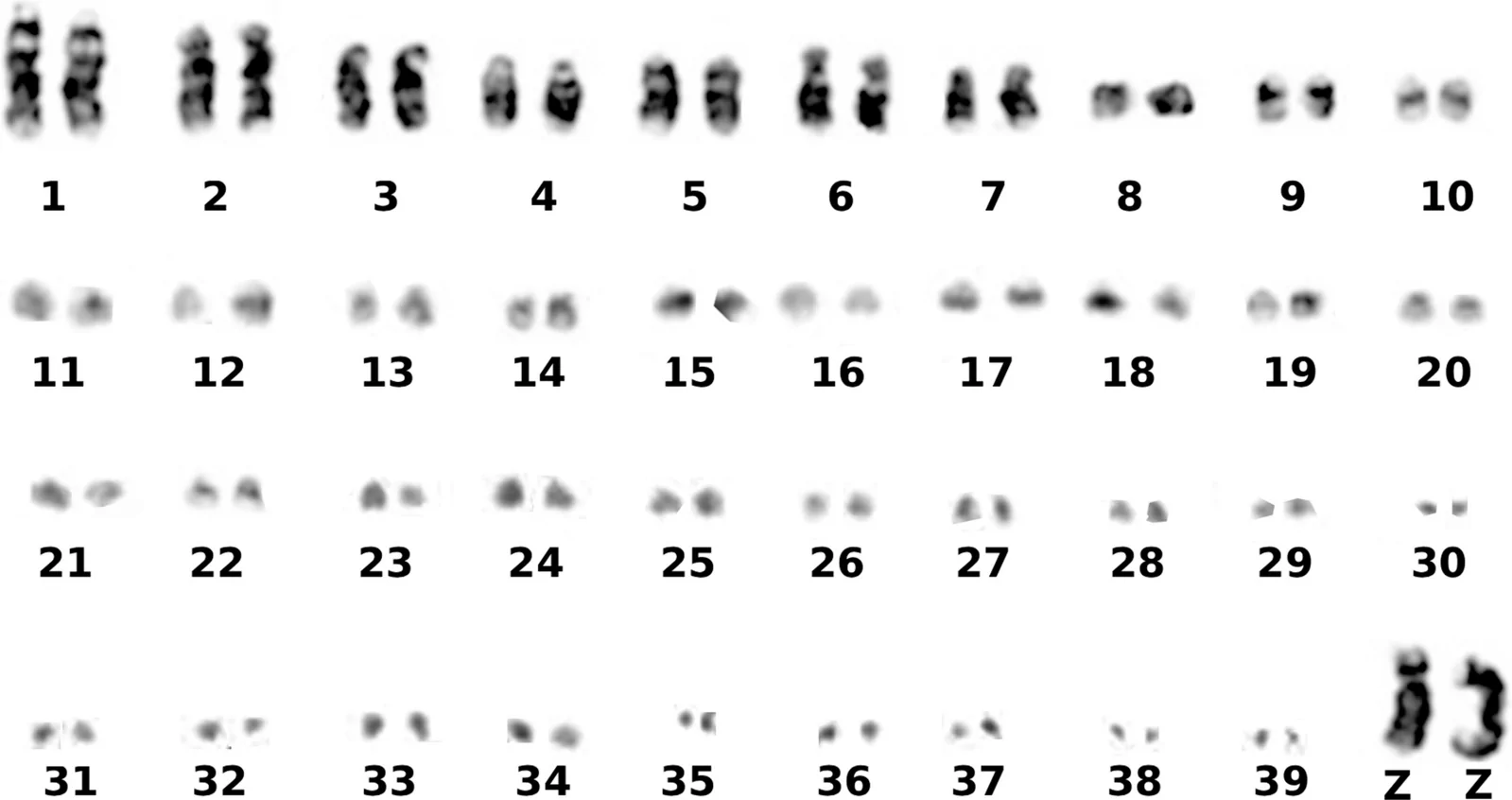
A G-banded karyotype of *Pyriglena leuconota*

*Pyriglena leuconota* possesses a karyotype of 2n = 80 and FN = 90. The macrochromosome set, composed of the first 13 pairs and the sex chromosome pair, includes eight telocentric pairs, five subtelocentric pairs, and 54 microchromosomes. The Z chromosome is submetacentric (Fig. 1).

**Fig. 2:**
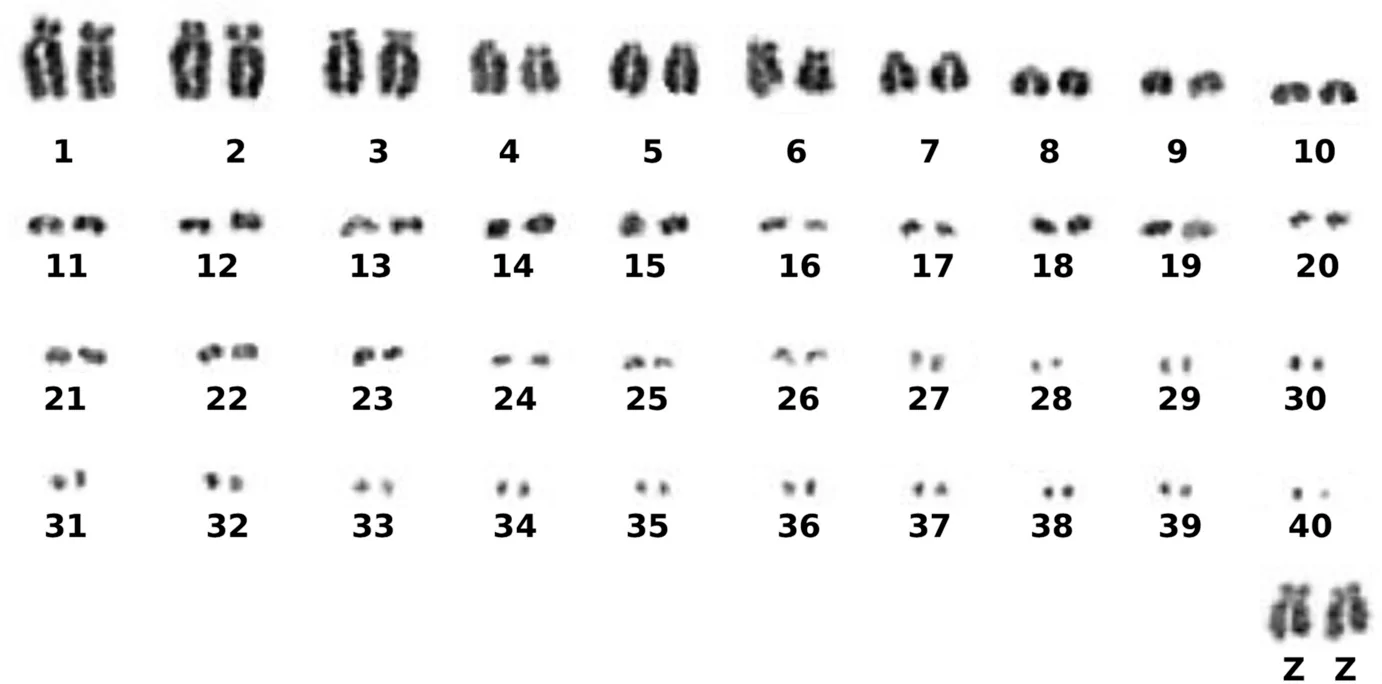
A conventional stained karyotype of *Thamnophilus aethiops*

Specimens of *Thamnophilus aethiops* exhibited a diploid number of 2n = 82 and NF = 92. The karyotype, based on macrochromosomes, includes eight telocentric pairs, five subtelocentric pairs, and 56 microchromosomes. The Z chromosome is submetacentric, while the W is telocentric (Fig. 2).

**Fig. 3:**
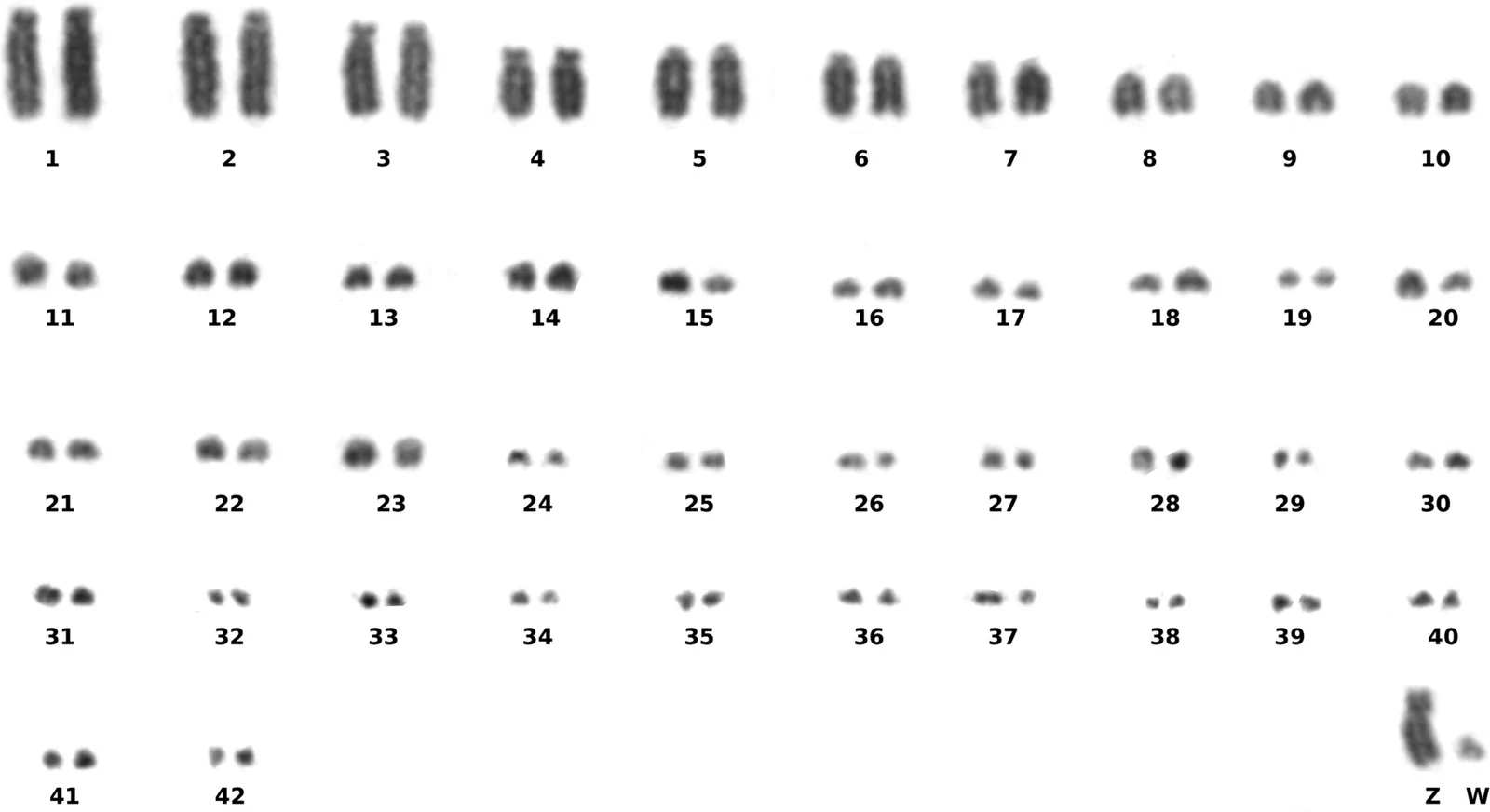
A conventional stained karyotype of *Phlegopsis nigromaculata*

*Phlegopsis nigromaculata* have a 2n = 86 and and FN = 96. Their macrochromosome sets include eight pairs of telocentric chromosomes, five pairs of subtelocentric chromosomes, and 60 microchromosomes. In both taxa, the Z and W chromosomes are subtelocentric (Fig. 3).

### Multidirectional chromosome painting

**Fig. 4:**
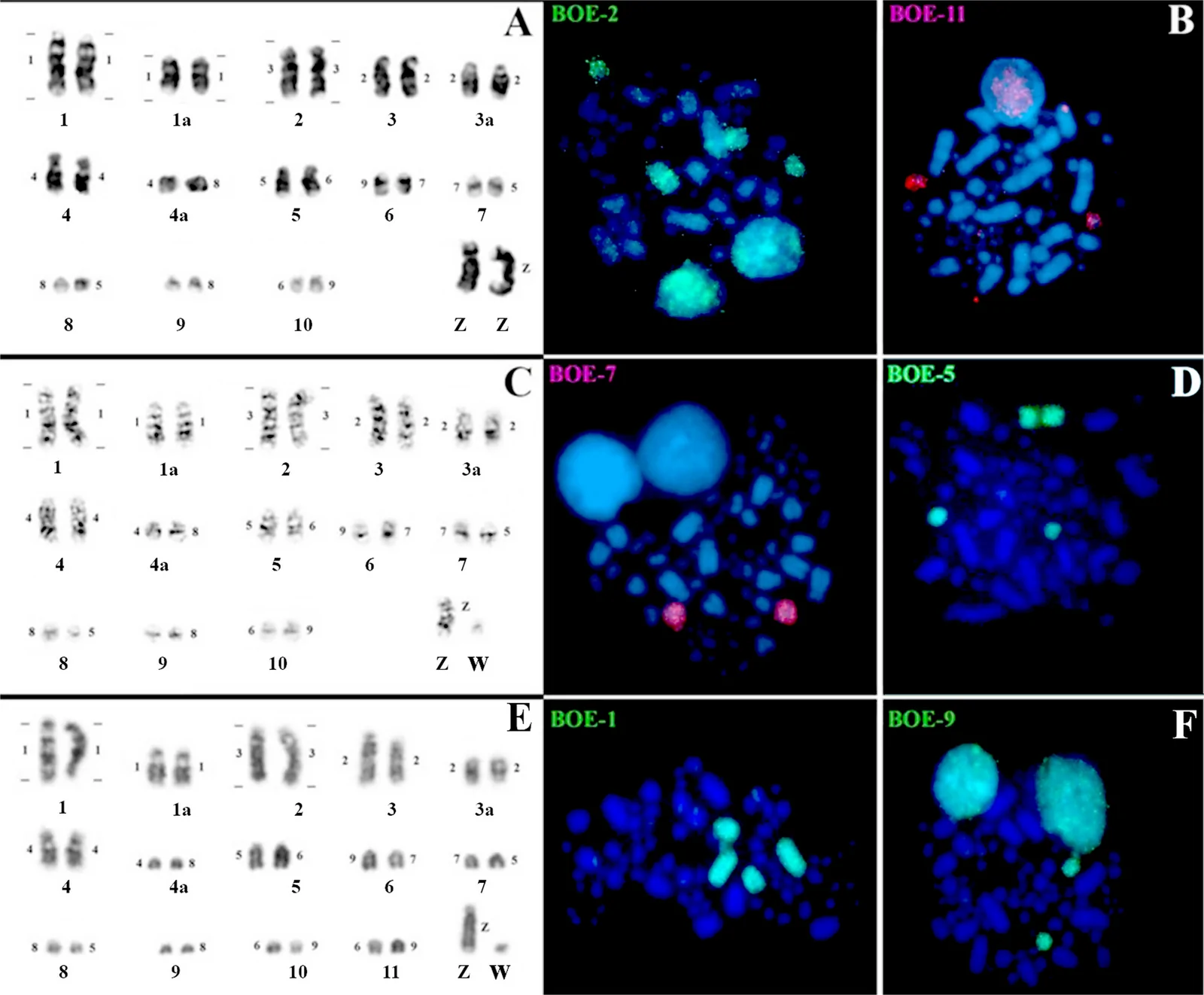
Left: Mapping of *Gallus gallus* (left) and *Burhinus oedicnemus* (right) probes. Right: Examples of chromosome painting on the analysed species with *Gallus gallus* (left) and *Burhinus oedicnemus* (right) probes. The probes were labelled with biotin; the green probe was detected with FITC; the red one with Cy3. DAPI was used as counterstaining. Each line represents one species. A and B: *Pyriglena leuconota*; C and D: *Thamnophilus aethiops*; E and F: *Phlegopsis nigromaculata*

*Pyriglena leuconota*: Whole chromosome painting using BOE probes identified 31 homologous segments within the PLE genome. Six probes (BOE-3, 4, 6, 7, 9, and Z) exhibited conserved synteny across PLE chromosomes, while the other probes produced two or more hybridization signals in PLE chromosomes (Figs. 4A, B).

*Thamnophilus aethiops*: Hybridization with BOE whole chromosome probes identified 31 homologous segments in the TAE genome. Six BOE probes (BOE-3, 4, 6, 7, 9, and Z) displayed synteny, each fully hybridizing with TAE chromosomes. The other probes generated two or more hybridization signals on the TAE chromosomes (Figs. 4C, D).

*Phlegopsis nigromaculata*: Hybridization using BOE whole chromosome probes detected 32 homologous segments within the PNI genome. Five BOE probes (BOE-3, 4, 6, 7, and Z) demonstrated conserved synteny in the PNI chromosomes, while the remaining probes produced one or more hybridization signals on the PNI chromosomes (Figs. 4E, F).

### Karyotypic description of *Myrmotherula longipennis*,* Myrmoborus myotherinus*, and *Thamnomanes caesius*

**Fig. 5:**
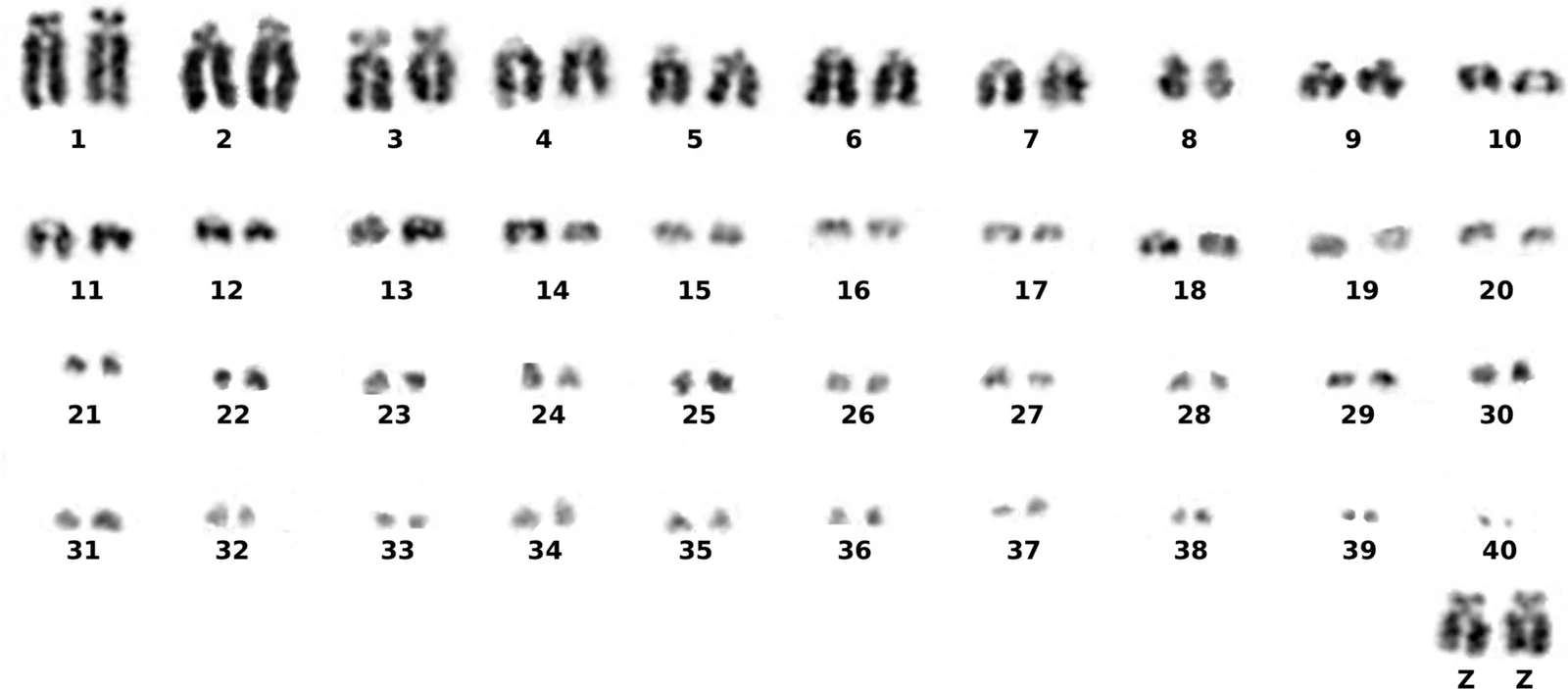
A conventional stained karyotype of *Myrmotherula longipennis*

**Fig. 6:**
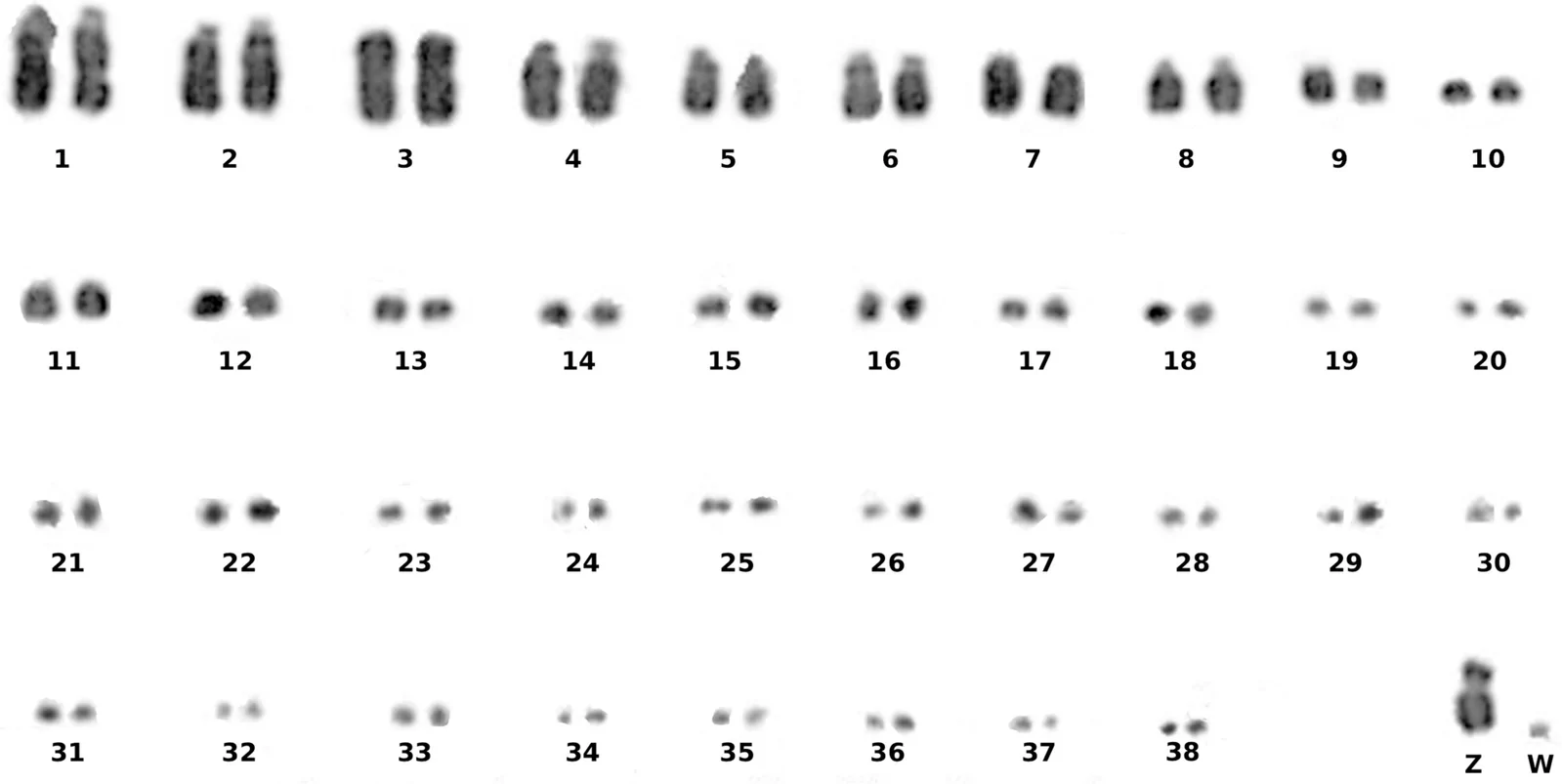
A A G-banded karyotype of *Myrmoborus myotherinus*

**Fig. 7:**
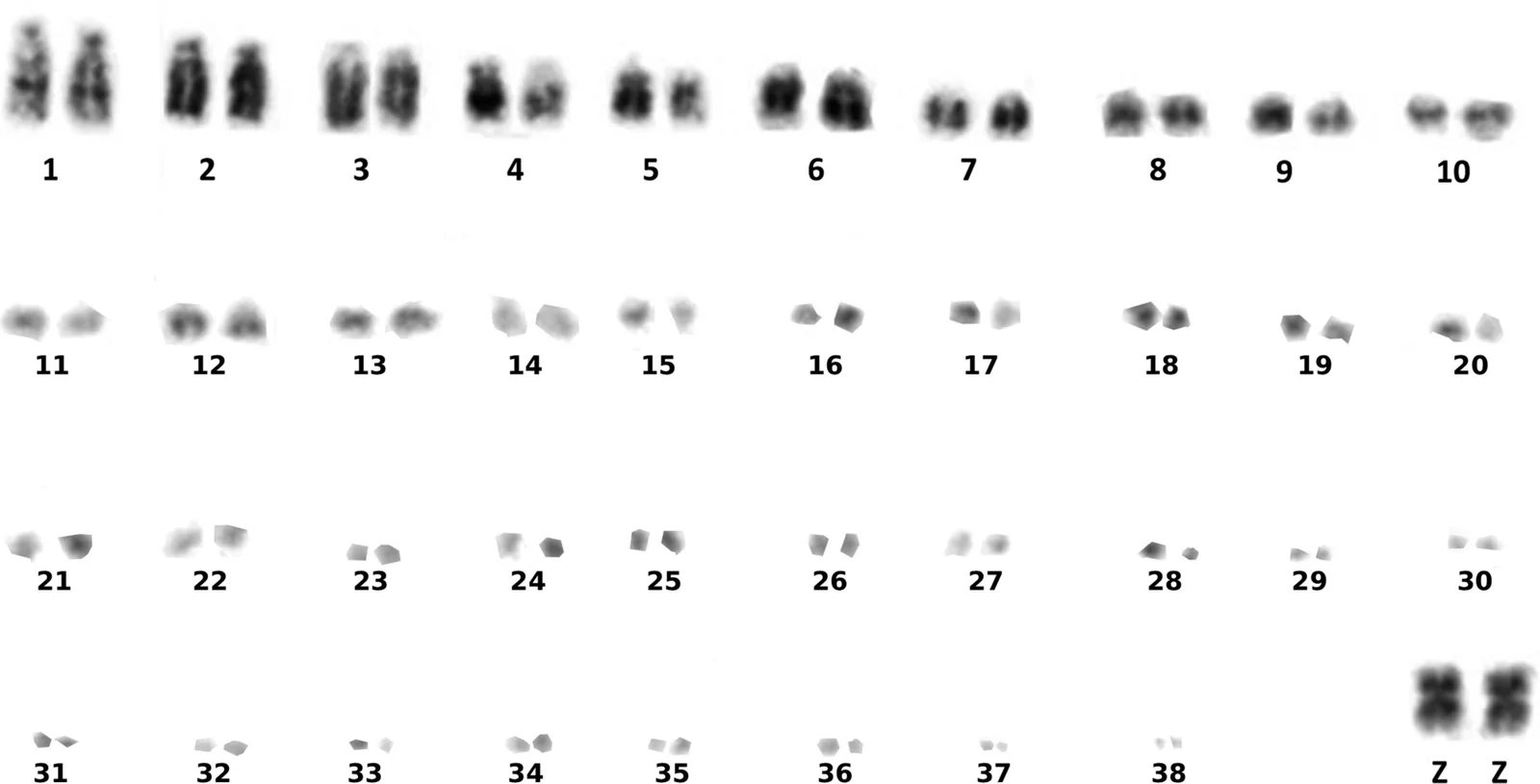
A G-banded karyotype of *Thamnomanes caesius*

*Myrmotherula longipennis* (MLO) displays 2n = 80 and FN = 88, with four subtelocentric pairs, seven telocentric pairs, and 58 microchromosomes; its Z chromosome is subtelocentric (Fig. 5)*. Myrmoborus myotherinus* (MMY) has a karyotype of 2n = 78 and FN = 86. The chromosomal composition includes three subtelocentric pairs, one metacentric pair, seven telocentric pairs, and 56 microchromosomes. The Z chromosome is submetacentric, while the W chromosome is acrocentric (Fig. 6). *Thamnomanes caesius* (TCA) features 2n = 78 and FN = 86, consisting of four subtelocentric pairs, six telocentric pairs, and 58 microchromosomes. The Z chromosome is metacentric (Fig. 7).

## Discussion

### Karyotypic diversity in Thamnophilidae birds

This study provides the first cytogenetic analysis of *Pyriglena leuconota* (2n = 80), *Thamnophilus aethiops* (2n = 82), and *Phlegopsis nigromaculata* (2n = 86), with samples collected from the Brazilian Amazon (see Figs. 1, 2, 3). The karyotypes observed conform to those typically found in Aves and Passeriformes, exhibiting a high diploid number, a limited number of macrochromosome pairs, and a substantial number of microchromosome pairs.

*Pyriglena leuconota* (PLE), with a diploid number of 2n = 80, exhibits a chromosome count distinct from its close relative *P. leucoptera* (PLU), which has 2n = 82 chromosomes—comprised of eight pairs of macrochromosomes and 33 pairs of microchromosomes (Ledesma et al. 2002). PLE is characterized by eight telocentric and five subtelocentric pairs, whereas PLU contains eight macrochromosome pairs, four subtelocentric and four telocentric. Both species share a subtelocentric Z chromosome. These observed differences likely result from pericentric inversions during chromosomal evolution and changes in diploid number associated with fission–fusion rearrangements. The discrepancy in karyotype arrangements between Ledesma et al. (2002), who reported only eight macrochromosome pairs, and our identification of 13 pairs may be due to the degree of chromosome constriction (with shorter chromosomes in the Ledesma karyotype) that could cause smaller macrochromosomes to appear as microchromosomes.

In addition to the genus *Pyriglena*, three other species within the Thamnophilidae family have been examined cytogenetically, with diploid numbers reported between 76 and 82. *Dysithamnus mentalis* (DME) exhibits a diploid number of 76, comprising eight pairs of macrochromosomes and 30 pairs of microchromosomes, all of which are subtelocentric. *Thamnophilus doliatus* (TDO) has a diploid number of 82, including eight pairs of macrochromosomes and 33 pairs of microchromosomes, with all macrochromosomes being telocentric. *Willisornis vidua* (WVI) shows 80 chromosomes, predominantly acrocentric, except for chromosomes 7 and 9, which are metacentric, along with 26 pairs of microchromosomes. The W and Z sex chromosomes are submetacentric (Ledesma et al. 2002; Lucca and Chamma 1977). The observed increase in diploid number is consistent with chromosomal fissions rather than fusions; however, other rearrangements such as pericentric inversions may also have occurred in the macrochromosomes, as indicated by classical cytogenetic studies (Hooper and Price 2015).

Morphological variation of the Z chromosome was observed among Thamnophilidae species included in this study. Specifically, the Z chromosome was subtelocentric in PNI and MLO, submetacentric in TAE, PLE, and WVI (Ribas et al. 2021), and metacentric in TCA (de Rosso et al. 2025). These differences are likely the result of chromosomal rearrangements, including pericentric inversions and/or centromeric repositioning (Nanda et al. 2008).

## Chromosome painting in Thamnophilidae and their relationship with another Passeriformes

Chromosome painting utilizing *Burhinus oedicnemus* and *Gallus gallus* probes (correlated with BOE probes, as reported by Nie et al. 2009) demonstrates that the majority of GGA chromosomes remain conserved *in toto* among Passeriformes, with the exception of GGA-1, 2, and 4. GGA-4 exhibits disruption across all examined Thamnophilidae species in this study, while GGA-6 is disrupted specifically in *Phlegopsis nigromaculata*. In TAE, GGA-1 is divided into two separate chromosomes (the first and second pairs, denoted as 1 and 1a), whereas GGA-2 manifests homology with the fourth and fifth pairs (3 and 3a) (Fig. 4). Additionally, GGA-4 is split into two distinct pairs (4 and 4a). These chromosomal arrangements were consistently observed in both *Phlegopsis nigromaculata* (PNI) and *Pyriglena leuconota* (PLE).

*Myrmotherula longipennis* has the first two chromosome pairs of nearly equal size, suggesting that GGA2 chromosome fission may be a general feature in Thamnophilidae species. In contrast, *M. myotherinus* has a third metacentric pair, which differs from other Thamnophilidae birds due to one pericentric inversion. This chromosome likely corresponds to GGA-2, and this configuration is considered a possible example of convergence within Thamnophilidae birds.

*Phlegopsis nigromaculata* possesses the highest diploid number (2n = 86 chromosomes) among Thamnophilidae species, exhibiting a karyotype that is slightly distinct from other Thamnophilinae birds described herein. Chromosomal painting demonstrates that BOE-11 divides into three segments, while BOE-9 (GGA-6) splits into two, patterns unique to *P. nigromaculata* (Fig. 4). These chromosomal rearrangements, along with fissions involving microchromosomes, account for the elevated diploid number observed in this species. Although both *P. nigromaculata* and *W. vidua* are classified within the tribe Pythyini (sensu), the karyotype of *Phlegopsis* more closely resembles that of *T. aethiops* from the Thamnophilini tribe. Notably, Pythyini species exhibit the most divergent karyotypes among Thamnophilinae. The karyotype of *W. vidua* markedly differs from those of other Passeriformes; chromosome painting reveals the fusion of *Burhinus oedicnemus* probes in three *W. vidua* chromosomes: BOE-9/11 (WVI-5), 11/6 (WVI-6), and micro/5 (WVI-7) (Ribas et al. 2021). These fusions likely involve the joining of ancestral macrochromosomes (GGA-6, GGA-5, and 7) with microchromosomes, considering the putative ancestral karyotype of Passeriformes (PAKP) proposed by Rodrigues et al. (2018; Table 2). As these rearrangements are absent in other Thamnophilidae or Passeriformes species, it can be inferred that such chromosomal modifications in *W. vidua* may have arisen independently, resulting in chromosomes unique to this species.

Furthermore, *in silico* analyses and FISH employing Bacterial Artificial Chromosome (BAC) probes have identified multiple rearrangements—including inversions and micro-inversions—within the avian genome. These structural changes are not detectable via chromosome painting alone, as illustrated in the antbird *W. vidua* (Skinner and Griffin 2012; Damas et al. 2016; Ribas et al. 2021). Integrating FISH with complementary methodologies offers enhanced resolution over chromosome painting alone, thereby facilitating the detection of intrachromosomal rearrangements previously unrecognized within this family.

**Fig. 8:**
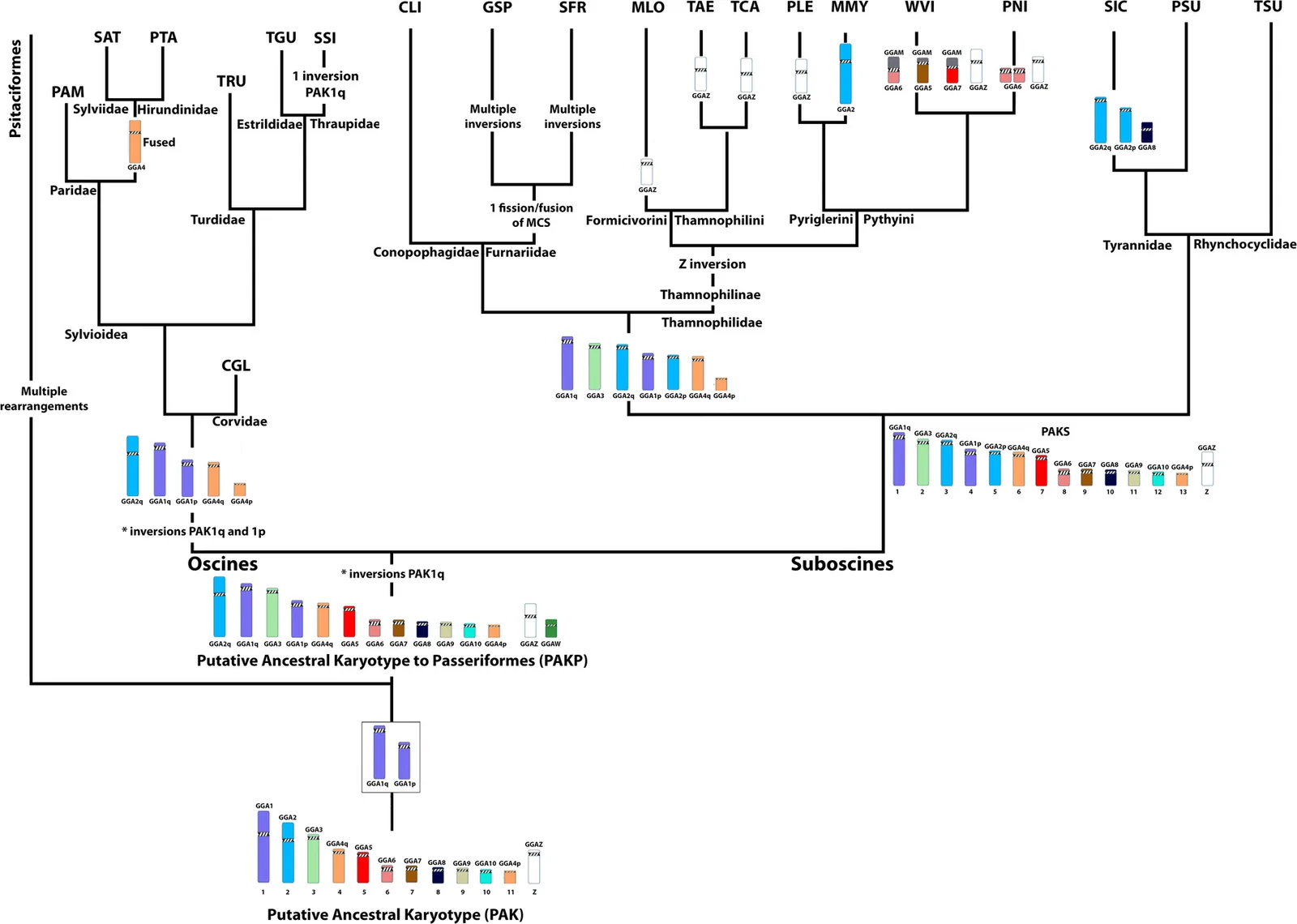
Partial reconstruction of chromosomal changes in Passeriformes birds based on Oliveros et al. (2019) and Moyle et al. (2009) phylogenies for family Thamnophilidae. The chromosome nomenclature followed Griffin et al. (2007), and refers to homology with GGA chromosomes. Chromosome data for Psittaciformes are from Furo et al. (2015); Oscines are from Nanda et al. (2011) (Paridae, *Parus major*, PAM; Sylviidae, *Sylvia atricapilla*, SAT), Barcellos et al. (2020) (Hirundinidae, *Progne tapera*, PTA), Kretchmer et al. (2014) (Turdidae, *Turdus rufiventris*, TRU), dos Santos et al. (2017) (Estrildidae, *Taeniopygia guttata*, TGU), and dos Santos et al. (2015) (Thraupidae, *Saltator similis*, SSI). Suboscines are from de Oliveira et al. 2020 (Conopophagidae, *Conopophaga lineata*, CLI), Ribas et al. (2018) (Furnariidae, *Glyphorynchys spirurus*, GSP), Krestchmer et al. (2018a) (Furnariidae, *Synallaxis frontalis*, SFR), present work (Thamnophilidae, *Myrmotherula longipennis*, MLO; *Thamnophilus aethiops*, TAE; *Thamnomaes caesius*, TCA; *Phlegopsis nigromaculata*, PNI), present work (Thamnophilidae, *Pyriglena leuconota*, PLE; *Myrmoborus myotherinus*, MMY), Ribas et al. (2021) (Thamnophilidae *Willisornis vidua,* WVI), Rodrigues et al. (2018) (Tyrannidae, *Satrapa icterophrys*, SIC, *Pitangus sulphuratus*, PSU), and Kretschmer et al. (2021a) (Rhynchocyclidae, *Tolmomyias sulphurescens*, TSU). PAKS = putative ancestral karyotype for Suboscines suggested here

To elucidate the dynamics of fusion and fission rearrangements in pairs corresponding to GGA-1, GGA-2, and GGA-4, the present analysis employed the family-level phylogeny for passerines proposed by Oliveros et al. (2019). By integrating karyotypic data obtained herein with previously published results from chromosome painting using whole chromosome probes for GGA, and mapping these karyotypes onto the established phylogeny, distinct patterns of chromosomal evolution were identified (Fig. 8). Within Suboscines, the study utilized data from Thamnophilidae, including *Thamnophilus aethiops, Phlegopsis nigromaculata*, *Pyriglena leuconota,* and *Myrmotherula longipennis* from this article, as well as *Willisornis vidua* from previous work (Ribas et al. 2021)*.* Additional data were drawn from Furnaridae (*Glyphorynchus spirurus* – Ribas et al. 2018; *Synallaxis frontalis* – Kretschmer et al. 2018a), Conopophagidae (*Conopophaga lineata* – de Oliveira et al. 2020), Tyrannidae (*Elaenia spectabilis* – Kretschmer et al. 2015; *Pitangus sulphuratus*, *Serpophaga subcristata*, *Satrapa icterophrys* – Rodrigues et al. 2018), and Rhynchocyclidae (*Tolmomyias sulphurescens* – Kretschmer et al. 2021a). In all evaluated species—except those from the last two families—the syntenic block corresponding to GGA-2 exhibited fission. In contrast, within Tyrannidae and Rhynchocyclidae, GGA-2 is typically fused, except in *Satrapa icterophrys*. Consequently, the fission of GGA-2 likely did not occur at the base of the Suboscines clade but rather on the branch leading to the families Thamnophilidae, Melanopareiidae, Conopophagidae, Grallariidae, Rhynocriptidae, Formicariidae, and Furnaridae (see Fig. 8). Detailed molecular analyses of Tyrannidae (Ohlson et al. 2008; Tello et al. 2009) indicate that *Satrapa icterophrys* belongs to the subfamily Fluviocolinae, tribe Xolmiini, which represents one of the most derived branches within Tyrannidae, whereas other studied taxa are more basal and retain the fused GGA-2 syntenic block. Accordingly, it is most parsimonious to interpret the fission of GGA-2 in *Satrapa icterophrys* as an autapomorphic characteristic of this species.

In Oscines, data pertaining to the families Hirundinidae (*Progne tapera*, *Progne chalybea*, *Pygochelidon cyanoleuca*—Barcellos et al. 2020), Turdidae (*Turdus rufiventris*, *Turdus albicollis*—Kretschmer et al. 2014), Estrildidae (*Taeniopygia guttata*—dos Santos et al. 2017), Fringillidae (*Serinus canaria*—dos Santos et al. 2017), Passerilidae (*Zonotrichia capensis*—Bü̈lau et al. 2018), Sylviidae (*Sylvia atricapilla*—Nanda et al. 2011), Corvidae (*Pica pica*, *Garrulus glandarius*—Nanda et al. 2011), Sittidae (*Sitta europaea*—Nanda et al. 2011), Paridae (*Parus major*, *Periparus ater*—Nanda et al. 2011), and Thraupidae (*Gubernatrix cristata*—Bü̈lau et al. 2021; *Saltator aurantiirostris*, *Saltator similis*—dos Santos et al. 2015; *Sicalis flaveola*—Kretschmer et al. 2021b) were reviewed. Across all species examined, GGA-2 remains in its non-fissioned state. With respect to the GGA-4 block, it is fissioned in all analyzed Oscine species except for those belonging to the Hirundinidae and Sylviidae families, where it is fused (Barcellos et al. 2020; Nanda et al. 2011). As both families are part of the same monophyletic branch within the superfamily Sylvioidea (Selvatti et al. 2015), this fusion likely represents a characteristic particular to this lineage. Although Paridae also falls under Sylvioidea, it belongs to a different branch that displays a fissioned GGA-4. Furthermore, the syntenic group corresponding to the fissioned GGA-4 is observed in superfamilies that diverged both prior to (Corvoidea) and after (Muscicapoidea, Passeroidea) Sylvioidea (Fig. 8).

Recent phylogenetic research has identified Psittaciformes and Passeriformes as sister groups, supported by both molecular and cytogenetic data (Hackett et al. 2008; Jarvis et al. 2014; Zhang et al. 2014). Accordingly, this order was selected as the outgroup for our analysis. Despite numerous chromosomal rearrangements within Psittaciformes, this group consistently exhibits fission of GGA-1 into two chromosome pairs. As highlighted by Kretschmer et al. (2018b), “the fission found in PAK1 (= GGA-1) in all species of Psittaciformes so far has also been detected in all Passeriformes studied by FISH, corroborating a recent proposal that Passeriformes and Psittaciformes are sister-groups.” For GGA-2, the syntenic block remains fused in most Psittaciformes examined, whereas GGA-4 is generally observed as fissioned. In *Amazona aestiva*, however, GGA-2 undergoes fission, while GGA-4 is fused (Nanda et al. 2007; Furo et al. 2015; Furo et al. 2018; Kretschmer et al. 2018b).

**Fig. 9:**
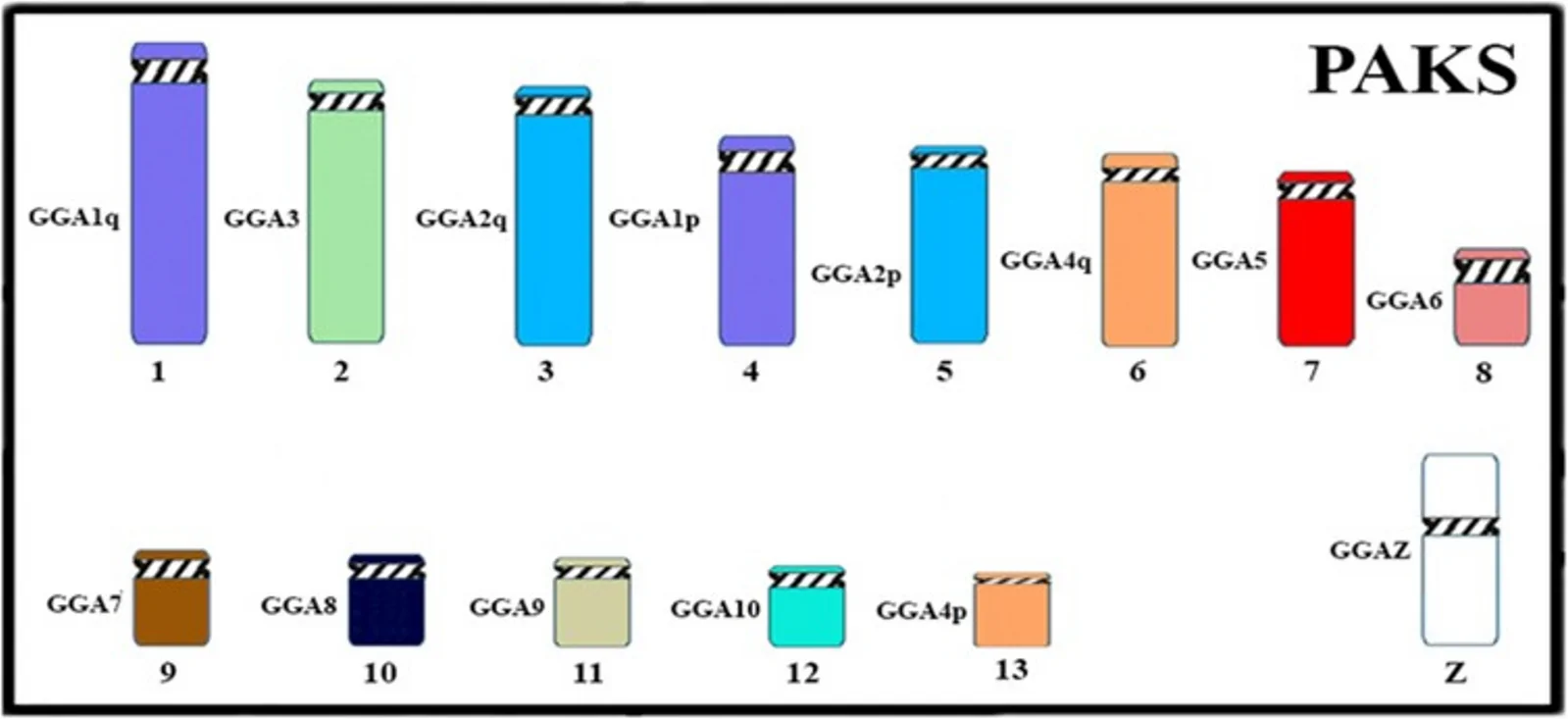
Partial putative ancestral karyotype of Passeriformes Suboscines, with homology to *Gallus gallus* chromosomes (GGA, indicated on the left)

We propose an ancestral karyotype for Suboscines featuring the fission of GGA-2, which may offer a more robust explanation for the karyotypic diversity observed among Passeriformes birds (see Figs. 8 and 9).

## Conclusions

This study presents the karyotypes and a chromosomal homology map comparing GGA and BOE in Thamnophilidae birds, revealing clade-specific patterns of genome evolution. These findings provide valuable markers for systematic research and demonstrate the utility of integrative cytogenetic and phylogenetic approaches. Through comparative analysis of the GGA-1, GGA-2, and GGA-4 syntenic blocks, our research elucidates the evolutionary dynamics of chromosomal rearrangements in both Suboscine and Oscine passerines, employing the phylogenetic framework of Oliveros et al. (2019) to map karyotypic traits onto molecular phylogeny. Most GGA chromosomes remain conserved *in toto* within Passeriformes, with exceptions observed for GGA-1, 2, 4, and 6 in *P. nigromaculata*. Furthermore, analysis using GGA probes across other Passeriformes families indicates that the centric fission of GGA2 is characteristic of Suboscines, emerging in the lineage leading to Thamnophilidae, Melanopareiidae, Conopophagidae, Grallariidae, Rhynocriptidae, Formicariidae, and Furnaridae.

## Data Availability

All data supporting the findings of this study are available within the article. The authors confirm that they followed the Brazilian laws implementing the Convention on Biological Diversity and Nagoya Protocol agreements.
